# Azelnidipine Inhibits Cultured Rat Aortic Smooth Muscle Cell Death Induced by Cyclic Mechanical Stretch

**DOI:** 10.1371/journal.pone.0102813

**Published:** 2014-07-17

**Authors:** Jing Zhao, Kentaro Ozawa, Yoji Kyotani, Kosuke Nagayama, Satoyasu Ito, Akira T. Komatsubara, Yuichi Tsuji, Masanori Yoshizumi

**Affiliations:** Department of Pharmacology, Nara Medical University School of Medicine, Kashihara, Nara, Japan; UT-Southwestern Med Ctr, United States of America

## Abstract

Acute aortic dissection is the most common life-threatening vascular disease, with sudden onset of severe pain and a high fatality rate. Clarifying the detailed mechanism for aortic dissection is of great significance for establishing effective pharmacotherapy for this high mortality disease. In the present study, we evaluated the influence of biomechanical stretch, which mimics an acute rise in blood pressure using an experimental apparatus of stretching loads in vitro, on rat aortic smooth muscle cell (RASMC) death. Then, we examined the effects of azelnidipine and mitogen-activated protein kinase inhibitors on mechanical stretch-induced RASMC death. The major findings of the present study are as follows: (1) cyclic mechanical stretch on RASMC caused cell death in a time-dependent manner up to 4 h; (2) cyclic mechanical stretch on RASMC induced c-Jun N-terminal kinase (JNK) and p38 activation with peaks at 10 min; (3) azelnidipine inhibited RASMC death in a concentration-dependent manner as well as inhibited JNK and p38 activation by mechanical stretch; and (4) SP600125 (a JNK inhibitor) and SB203580 (a p38 inhibitor) protected against stretch-induced RASMC death; (5) Antioxidants, diphenylene iodonium and tempol failed to inhibit stretch-induced RASMC death. On the basis of the above findings, we propose a possible mechanism where an acute rise in blood pressure increases biomechanical stress on the arterial walls, which induces RASMC death, and thus, may lead to aortic dissection. Azelnidipine may be used as a pharmacotherapeutic agent for prevention of aortic dissection independent of its blood pressure lowering effect.

## Introduction

With the rapid progress of population aging in most developed countries, the number of patients with atherosclerosis has remarkably increased; this is becoming an extremely serious problem requiring urgent attention [1], [2]. Among cardiovascular diseases, acute aortic dissection presents with sudden onset of severe pain and a high fatality rate [3], [4]. It has been reported that various endovascular techniques with minimally invasive characteristics have been applied extensively to elderly patients and have proven to be effective in acute aortic dissection treatment. However, most successful cases to date have been restricted to surgical operations, and there is little evidence relating to effective drug treatment or pharmacotherapy.

It is well recognized that aortic dissection occurs when a small tear generated in the inner aortic wall extends along the wall of the aorta and causes blood to flow between the layers of the tunica media and adventitia of the aorta, forcing the layers apart. Despite the pathophysiological interpretation, the detailed mechanism for aortic dissection still remains unclear. Various efforts have been recently made to clarify the possible reasons for aortic dissection. Collins et al. reported that progressive loss of smooth muscle cells is observed in the specimens of acute aortic dissection characterized by aortic medial degeneration [5]. Wernig et al. and Chen et al. confirmed that mechanical stretch can induce apoptosis in vascular smooth muscle cells (VSMCs) [6], [7]. Hipper and Isenberg found that cyclic mechanical strain reduced DNA synthesis in VSMCs [8]. Along with these findings, we hypothesized that acute mechanical stretching force, which mimics an acute rise in blood pressure, may cause rat aortic smooth muscle cell (RASMC) death including apoptosis, thus leading to the occurrence of aortic dissection.

Azelnidipine has been approved for the treatment of patients with hypertension and is extensively used in developed countries [9]–[11]. Many researchers considered that the effects of azelnidipine can be attributed primarily to its protection of cardio-renal functions by means of lowering blood pressure. Kondo et al. and Fujimoto et al. reported that azelnidipine had protective effects on renal injury induced by angiotensin II infusion through improvement in renal microcirculation [12], [13]. In addition, it was reported that azelnidipine imparted antihypertensive effects and prevented cardiac hypertrophy in the Spontaneously Hypertensive Rat [14] and improved contractile dysfunction in stunned myocardium in dogs [15]. However, most of these studies have only emphasized the protective effects of azelnidipine on cardio-renal functions through lowering of blood pressure, and there have been almost no findings on the pathophysiological mechanism by which azelnidipine protects against progression of acute aortic dissection. Based on the findings mentioned above, we hypothesized that azelnidipine, in addition to its blood pressure lowering effect, may inhibit VSMC death (including apoptosis) and thereby reduce the occurrence of aortic dissection.

In the present study, we used an experimental apparatus of stretching loads in vitro that can simulate sudden increases of blood pressure and observed RASMC death induced by biomechanical stretch. Furthermore, we investigated whether azelnidipine inhibited stretch-induced VSMC death. The effect of azelnidipine on changes in intracellular signaling by biomechanical stretch was also examined to provide a possible mechanism by which azelnidipine may be used as a pharmacotherapeutic agent for the prevention of aortic dissection independent of its blood pressure lowering effect.

## Materials and Methods

### Cell culture and mechanical stretch

The study design was approved by an ethics review board of guidelines for the use of laboratory animals of Nara Medical University (No. 11011) and this study conducted in accordance with the guide for the Care and Use of Laboratory Animals as adopted and promulgated by the United States National Institutes of Health.

RASMCs were isolated from the thoracic aorta of 8-week-old male Sprague–Dawley rats by enzymatic digestion, as previously described [16]. Cells were grown in Dulbecco’s modified Eagle’s medium (DMEM, Sigma-Aldrich, St Louis, MO) supplemented with 10% fetal bovine serum (FBS, HyClone, Logan, UT), penicillin (100 U/mL, Invitrogen, Carlsbad, CA), and streptomycin (100 µg/mL, Invitrogen) at 37°C under 5% CO_2_ in a humidified incubator. RASMCs were used for experiments between the third and sixth passages. The cells were cultured in collagen I-coated (70 µg/cm^2^) silicon chambers (STREX Inc, Osaka, Japan). When the cell confluency in culture was estimated to be 70–80%, the medium was replaced with unsupplemented DMEM. The cells were further cultured for 24 h and then subjected to cyclic mechanical stretch (60 cycles/min, 15% elongation) for a given time period using the computer-controlled mechanical Strain Unit (STREX Inc, Osaka, Japan). After cyclic stretch, the medium was replaced with DMEM-containing 0.1% FBS. For western blot analysis, a portion of the RASMCs were lysed immediately after stretch stimulation and lysate proteins were collected in the manner described earlier [17]. In addition, a portion of the RASMCs were further incubated for 24 h to detect cell viability by MTT assay and cell death by the release of lactate dehydrogenase (LDH). In some experiments, RASMCs were pre-incubated with azelnidipine and mitogen-activated protein (MAP) kinase inhibitors (SP600125 or SB203580) 20 min prior to stimulation with cyclic mechanical stretch. Azelnidipine (CS905), SP600125, and SB203580 are abbreviated as CS, SP, and SB in figures. Band intensities were quantified by densitometry of the immunoblots using NIH Image J software. The values of phospho-MAP kinase have been normalized to total MAP kinase measurements and then expressed as the ratio of normalized values to protein in the control group as 1 (n = 3 per group).

### Materials

Materials were purchased from Wako (Kyoto) or Nacalai Tesque (Kyoto) unless stated otherwise. Azelnidipine (CS905) was from Daiichi Sankyo, Inc (Osaka). The antibodies used for western blot analyses were as follows: anti-phospho-SAPK/JNK (Thr183/Tyr185) antibody and anti-phospho-p38 MAP kinase (Thr180/Tyr182) antibody were purchased from Cell Signaling Technology, while ECL and ECL plus systems were purchased from GE Healthcare. Collagen I was purchased from Nippon Meat Packers, Inc. (Osaka). All chemical compounds were dissolved in dimethyl sulfoxide (DMSO) at final concentration less than 1% except for special notification.

### Statistical analysis

All experimental values were expressed as mean ± standard deviation. Analysis of variance along with subsequent Student’s *t*-test was used to determine significant differences in multiple comparisons. A *P* value<0.05 was considered to be significant.

## Results

### Effects of cyclic mechanical stretch on cell viability in RASMCs

**Figure 1:**
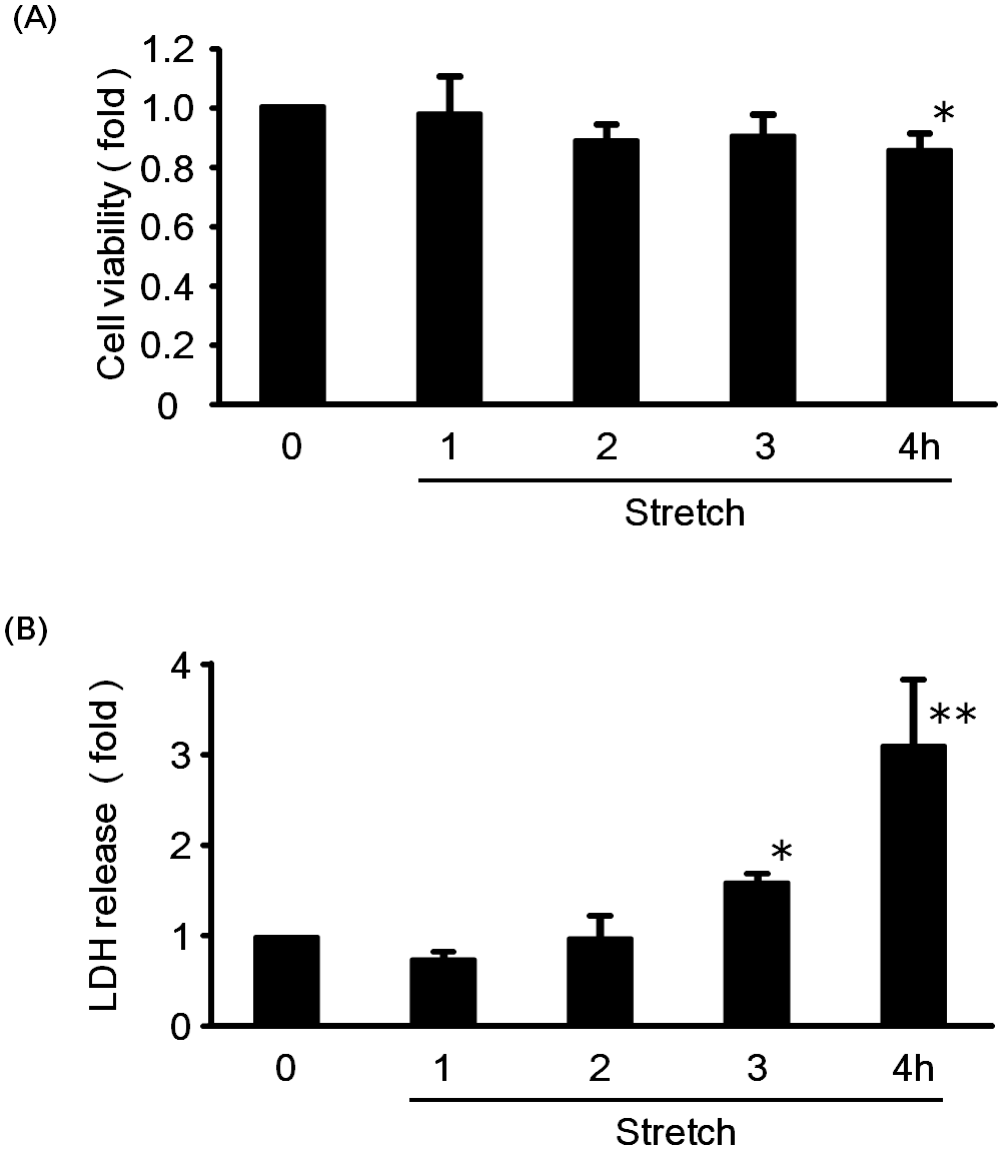
Time course for the effects of cyclic mechanical stretch (15% elongation) on cell viability (A) and cell death (B) in RASMCs up to 4 h. The cells cultured under standard conditions were exposed to cyclic mechanical stretch by 15% elongation for various time periods (from 1 to 4 h) and then incubated for 24 h. Cell viability and cell death were evaluated by MTT assay and the release of lactate dehydrogenase (LDH), respectively. Colorimetric analysis of each value was normalized by arbitrarily setting the absorbance value of the control cells (Ctrl.) to 1. Each value represents the mean ± standard deviatin (S.D.) (n = 4). The asterisks represent significant differences compared with the control value (*P<0.05).

The effect of cyclic mechanical stretch on the viability of RASMCs was firstly examined by measuring MTT reduction and LDH released. Figures 1A and 1B show the viability and death rate (reflected by LDH released to the medium) of RASMCs subjected to cyclic mechanical stretch by 15% elongation for various time periods, respectively. It was observed that viability was reduced with an increase in stretch time; the viability of RASMCs stimulated for 4 h decreased by 14% as compared with those of untreated cells. In the meantime, the death rate of RASMCs increased nearly three-fold with increase in stretch time from 1 h up to 4 h. These results suggest that cyclic mechanical stretch induced cell death in RASMCs.

### Cyclic mechanical stretch induced the activation of MAP kinases in RASMCs

**Figure 2:**
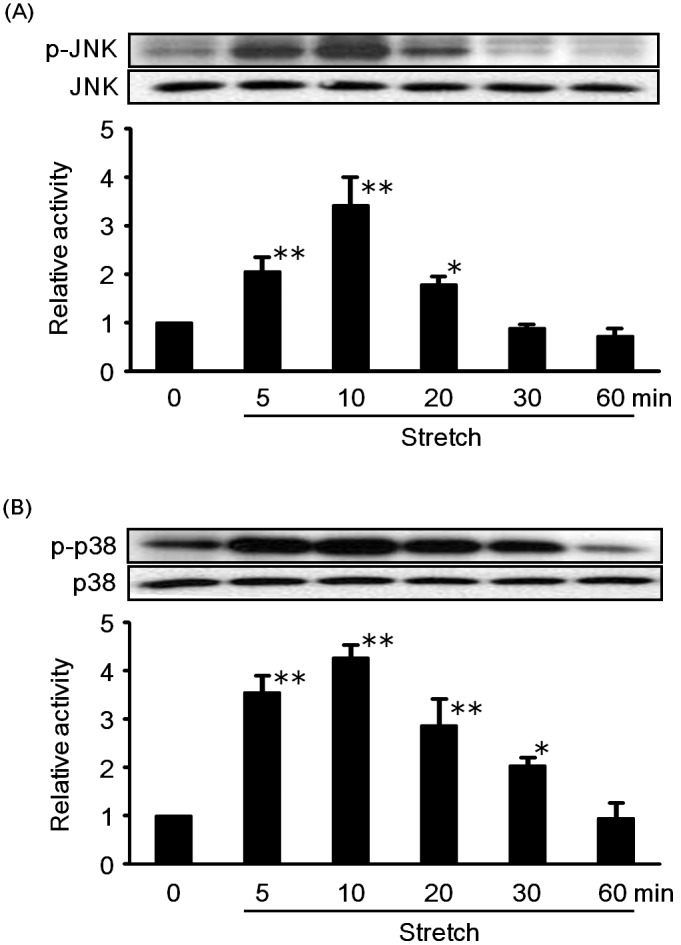
Time course for the effects of cyclic mechanical stretch (15% elongation) on the activation of JNK (A) and p38 (B) in RASMCs. The cells cultured under standard conditions were exposed to cyclic mechanical stretch by 15% elongation for various time periods (from 5 min to 60 min). The phosphorylation of JNK (A) and p38 (B) were measured as described under *Materials and Methods*. Densitometric analysis of each value was normalized by arbitrarily setting the densitometric value of the control cells (Ctrl.) to 1. Each value represents the mean ± S.D. (n = 3). The asterisks represent significant differences compared with the control value (*P<0.05, **P<0.01).

The effects of cyclic mechanical stretch on the activation of JNK and p38 (members of MAP kinases family proteins) were assessed by western blot analysis (Figure 2). RASMCs were exposed to cyclic mechanical stretch with a 15% elongation for different periods of time, and the phosphorylation of JNK (A) and p38 (B) was measured. As shown in Figures 2A and 2B, both JNK and p38 in RASMCs were activated by cyclic mechanical stretch. For both JNK and p38, the extent of activation increased with increase in stretch time, reaching a peak at 10 min and then gradually decreasing to basal level with further increasing stretch time up to 60 min. These findings imply that the activation of JNK and p38 seemed to be involved in was well as influence RASMCs death. The results obtained here are in agreement with those reported earlier in the literature [18], [19].

In order to clarify the possible mechanisms of how cyclic mechanical stretch influences cell death, the following two experiments were undertaken.

### Azelnidipine inhibited cyclic mechanical stretch-induced JNK and p38 MAP kinase activation in RASMCs

**Figure 3:**
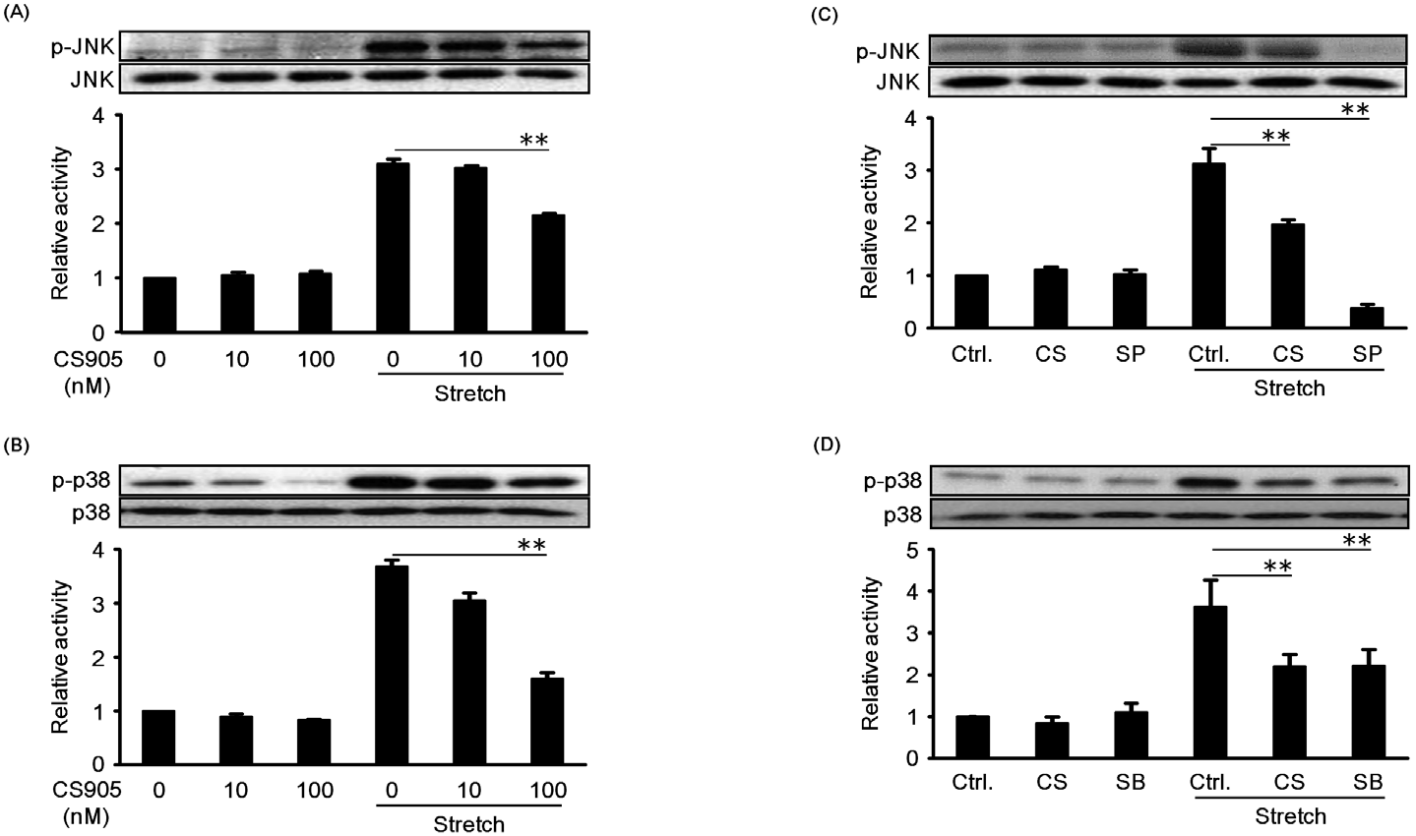
Effects of different concentrations of azelnidipine on the activation of JNK (A) and p38 (B) induced by cyclic mechanical stretch in RASMCs; and the comparison of the effects of azelnidipine and MAP kinase inhibitors on the activation of JNK (C) and p38 (D) induced by cyclic mechanical stretch in RASMCs. The cells were pre-incubated by CS905 (10 nM, 100 nM), SP600125 (20 µM), and SB203580 (20 µM) for 20 min prior to exposing to cyclic mechanical stretch by 15% elongation for 10 min. The phosphorylation of JNK and p38 were measured as described under *Materials and Methods*. Azelnidipine (CS905), SP600125, and SB203580 are abbreviated as CS, SP, and SB. Densitometric analysis of each value was normalized by arbitrarily setting the densitometric value of the control cells (Ctrl.) to 1. Each value represents the mean ± S.D. (n = 3). The asterisks represent significant differences compared with the stretched control value (*P<0.05, **P<0.01).

The effects of azelnidipine on cyclic mechanical stretch-induced activation of JNK and p38 in RASMCs were firstly examined and the results are shown in Figures 3A and 3B, respectively. In Figures 3C and 3D, we compared the effects of azelnidipine and MAP kinase inhibitors on cyclic mechanical stretch-induced activation of JNK and p38 in RASMCs, respectively. It was obvious that JNK and p38 MAP kinase activation were significantly attenuated by azelnidipine in a dose-dependent manner. Both JNK and p38 activation induced by cyclic mechanical stretch were inhibited by their respective inhibitors (SP600125 and SB203580), implying that the inhibition of JNK and p38 activation could be beneficial to suppressing mechanical stretch-induced RASMC death.

### Cyclic mechanical stretch-induced cell death was inhibited by azelnidipine and MAP kinase inhibitors in RASMCs

**Figure 4:**
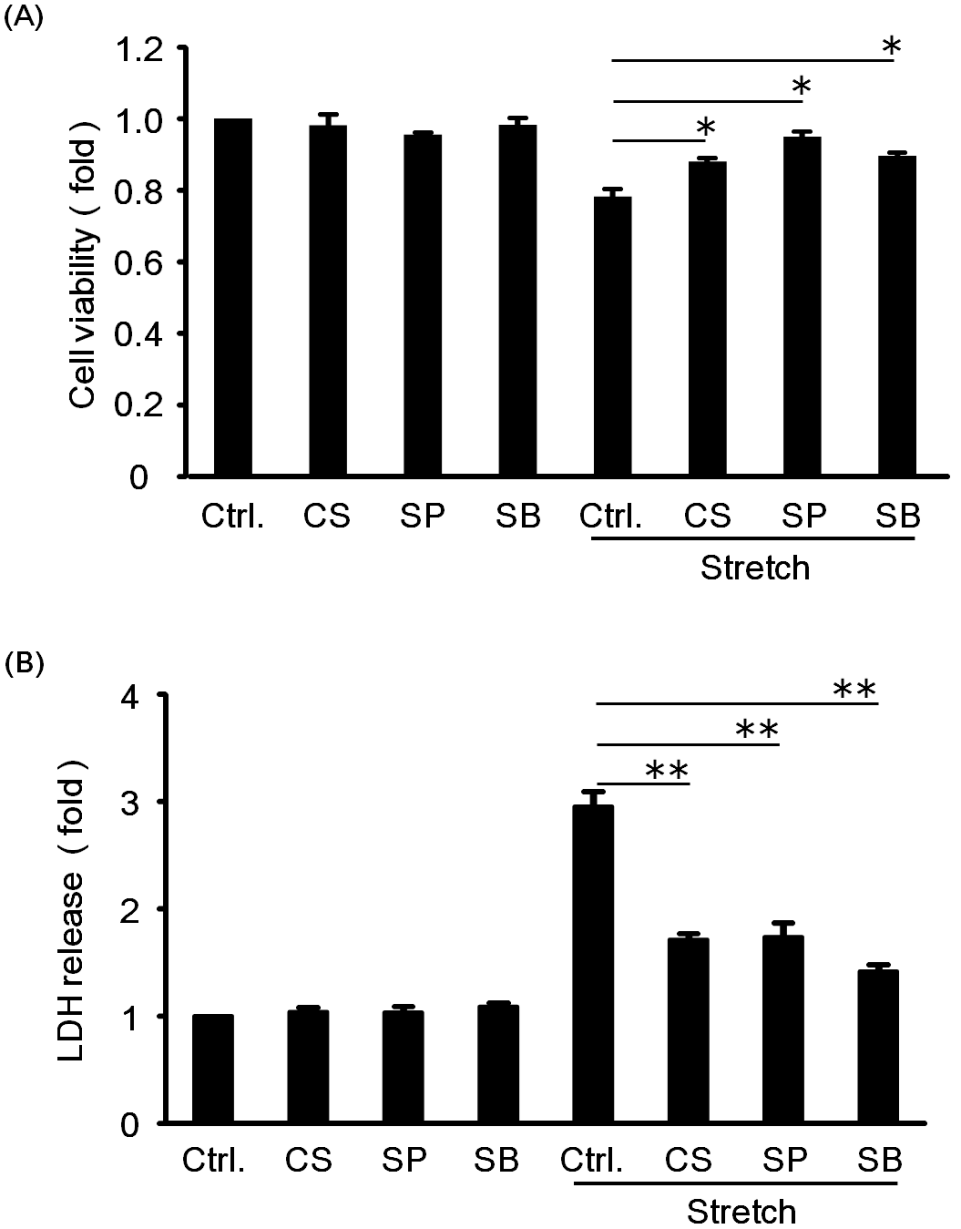
Comparison of the cell viability (A) and LDH release (B) induced by cyclic mechanical stretch in RASMCs with or without azelnidipine or MAP kinase inhibitors. The cells were pre-incubated by CS905 (100 nM), SP600125 (20 µM), and SB203580 (20 µM) for 20 min prior to exposing to cyclic mechanical stretch by 15% elongation for 4 h and then incubated for 24 h. Cell viability and cell death were evaluated by MTT assay and the release of lactate dehydrogenase (LDH), respectively. Azelnidipine (CS905), SP600125, and SB203580 are abbreviated as CS, SP, and SB. Colorimetric analysis of each value was normalized by arbitrarily setting the absorbance value of the control cells (Ctrl.) to 1. Each value represents the mean ± S.D. (n = 4). The asterisks represent significant differences compared with the stretched control value (*P<0.05, **P<0.01).

Figure 4A compares the relative cell viability for RASMCs in culture media with or without azelnidipine or MAP kinase inhibitors. It was found that azelnidipine, SP600125, and SB203580 all significantly increased the viability of RASMCs. Figure 4B compares the LDH released from the RASMCs into the culture media with or without azelnidipine or MAP kinase inhibitors. Compared with the positive control, azelnidipine, SP600125, and SB203580 significantly reduced the death rate of RASMCs. These results indicate that azelnidipine and MAP kinase inhibitors potentially inhibit RASMC death induced by cyclic mechanical stretch.

### Effects of antioxidants, diphenylene iodonium (DPI) and tempol on cyclic mechanical stretch-induced cell death

**Figure 5:**
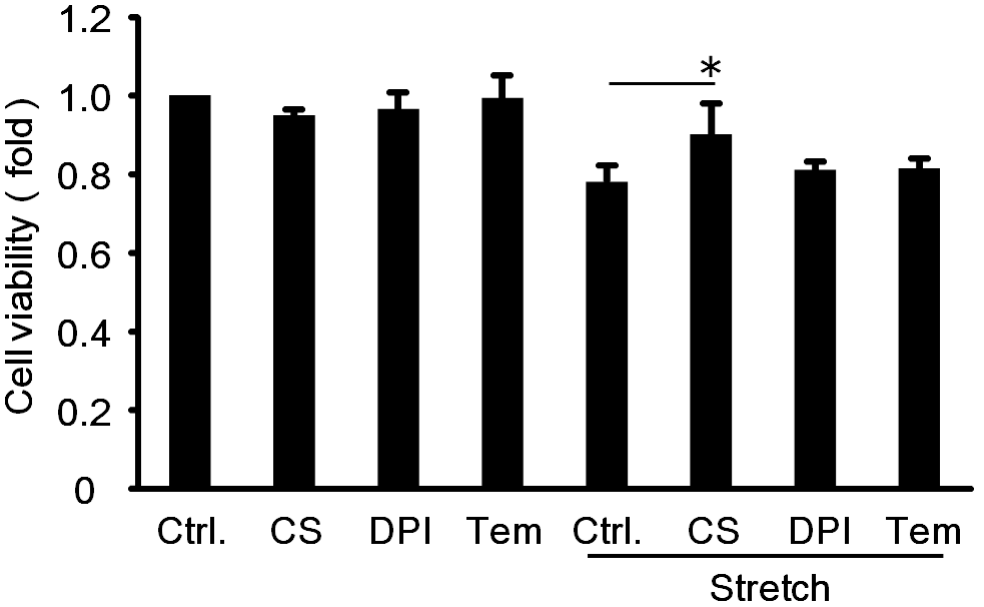
Effects of antioxidants on cyclic mechanical stretch-induced RASMC death. The cells were pre-incubated by CS905 (100 nM), diphenylene iodonium (5 µM), and tempol (1 µM) for 20 min prior to exposing to cyclic mechanical stretch by 15% elongation for 4 h and then incubated for 24 h. Cell viability was evaluated by MTT assay. Azelnidipine (CS905), diphenylene iodonium, and tempol are abbreviated as CS, DPI, and Tem. Colorimetric analysis of each value was normalized by arbitrarily setting the absorbance value of the control cells (Ctrl.) to 1. Each value represents the mean ± S.D. (n = 4). The asterisks represent significant differences compared with the stretched control value (*P<0.05, **P<0.01).

It has been reported that azelnidipine has the effects of anti-inflammation and antioxidant in mouse aneurysmal models [20], [21]. Therefore, we next examined the effects of antioxidants, DPI and tempol on cyclic mechanical stretch-induced RASMC death. As shown in Figure 5, pretreatment with both DPI and tempol failed to inhibit mechanical stretch-induced RASMCs death, suggesting that oxidative stress may not be involved in RASMC death induced by cyclic mechanical stretch.

## Discussion

The major findings of the present study are as follows: (1) cyclic mechanical stretch of RASMC caused cell death in a time-dependent manner up to 4 h; (2) cyclic mechanical stretch of RASMCs induced JNK and p38 activation with peaks at 10 min; (3) azelnidipine, a calcium channel blocker, inhibited the activation of JNK and p38 by cyclic mechanical stretch in a concentration-dependent manner; and (4) azelnidipine and JNK or p38 inhibitors protected against stretch-induced RASMC death; (5) Antioxidants, DPI and tempol failed to inhibit stretch-induced RASMC death.

In this work, we recall the assumption that acute biomechanical stretch applied to cultured VSMCs in vitro simulating a sudden increase in blood pressure resulted in VSMC death that led to aortic dissection. As shown in Figure 1A, we found that cyclic mechanical stretch caused cell death of RASMCs in a time-dependent manner. The cell fate also can be supported by the fact that LDH release from the cells was increased (Fig. 1B). This implies that RASMC death induced by rapidly developed biomechanical stretch is one of the likely reasons for aortic dissection. Some other researchers have also reported that stretching loads induce smooth muscle cell death, which is consistent with the present study [6], [7], [22], [23]. On the other hand, it has been reported that cyclic mechanical stretch of cells results in cell proliferation [23], [24]. Such a phenomenon was also observed as we applied mechanical stretch to RASMCs in vitro for 24 h using a stretching apparatus (data not shown here). In our experimental conditions, cell death occurred after stretch stimulation for 4 h and subsequently surviving cells entered into a proliferation cycle, showing a gradual increase in cell numbers that might be higher than that of the control at the end of 24 h as a result of growth and division. From the above findings, we concluded that mechanical stretch led to both cell death and cell proliferation. It appeared that the extent and duration of mechanical stretch decided what would happen to those SMCs in vitro. Our experimental results indicated that acute mechanical stretch primarily contributed to SMC death.

Azelnidipine is a calcium channel antagonist (blocker) that has been applied extensively to the treatment of patients with hypertension all over the world. In the present study, we found that azelnidipine inhibited RASMC death induced by cyclic mechanical stretch (Fig. 4A). Under the present conditions, the protective effects of azelnidipine on RASMCs seemed to be different from its antihypertensive effects, because the cyclic mechanical stretch was applied. It has been reported that azelnidipine exhibited a suppressing effect on aneurysm development in mouse models of aortic aneurysms, which was thought to be independent of its antihypertensive action [20], [21]. Those researchers considered that azelnidipine suppressed the progression of aortic aneurysm through both anti-inflammatory [20] and antioxidant mechanisms [21]. Although the exact mechanisms are unresolved, our findings suggest that the preventive effects of azelnidipine against aortic aneurysm should be associated with its inhibitory effect of RASMCs death induced by cyclic mechanical stretch, apart from its antihypertensive effect. Such an assumption needs to be further confirmed by examining the fate of SMCs using an in vivo model of acute aortic dissection and may be a topic for future research. In addition, attention should also be paid to other calcium channel blockers in future with the aim of comparing their effects on stretch-induced RASMC death.

Among MAP kinases, JNK and p38 were recognized to be related to cell death or apoptosis [25]–[27]. Our experimental data demonstrated that JNK and p38 in RASMCs were activated by cyclic mechanical stretch (Fig. 2). Cheng et al. also reported that JNK activation was involved in mechanical stretch-induced VSMC death [7]. Yoshimura et al. found that JNK played a significant role in the formation and development of aortic aneurysm [28]. Similarly, some researchers reported that mechanical stretch led to p38 activation [18], which is in agreement with our results. Actually, we found that cyclic mechanical stretch-induced RASMC death was suppressed when the activity of JNK and p38 was inhibited by their inhibitors (Fig. 4). These findings indicated that JNK and p38 activation is likely to be associated with cyclic mechanical stretch-induced RASMC death. Since azelnidipine inhibited both JNK and p38 activation by cyclic mechanical stretch, it can be assumed that azelnidipine prevented cyclic mechanical stretch-induced RASMC death through inhibition of JNK and p38 activation in RASMCs.

We have reported in the previous study that p38 and JNK are oxidative stress sensitive [29]. Ohyama et al. also reported that azelnidipine has an effect of antioxidant in mouse aneurysmal models [21]. Therefore, it is conceivable that azelnidipine inhibited cyclic mechanical stretch-induced cell death through inhibiting JNK and p38 activation via its anti-oxidative mechanisms. In order to clarify this point, we performed additional experiments of cyclic mechanical stretch-induced RASMC death using antioxidants, diphenylene iodonium and tempol. As shown in Fig. 5, pretreatment with these anti-oxidants did not affected the relative cell viability of RASMCs, suggesting that the inhibiting effects of azelnidipine on stretch-induced cell death could not be attributed to its anti-oxidative effect.

In conclusion, azelnidipine inhibited RASMC death induced by acute cyclic mechanical stretch (originating from a simulated increase in blood pressure in vitro). JNK and p38 in RASMCs were activated by cyclic mechanical stretch; however, the activation was inhibited by azelnidipine. Similar to azelnidipine, pharmacological inhibition of JNK and p38 activation by mechanical stretch suppressed cyclic mechanical stretch-induced RASMC death. It is expected that the mechanism of acute aortic dissection will be clarified from further study of the fate of VSMCs by acute cyclic mechanical stretch. Azelnidipine may be an alternative candidate for prevention of acute aortic dissection independent of its blood pressure lowering effect.
